# Fabrication of All-Solid Organic Electrochromic Devices on Absorptive Paper Substrates Utilizing a Simplified Lateral Architecture

**DOI:** 10.3390/ma13214839

**Published:** 2020-10-29

**Authors:** Guodong Liu, Yu Liu, Meiyun Zhang, Fredrik Pettersson, Martti Toivakka

**Affiliations:** 1 College of Bioresources Chemical and Materials Engineering, Shaanxi University of Science and Technology, Xi’an 710021, China; yuliu.410@aliyun.com; 2 National Demonstration Center for Experimental Light Chemistry Engineering Education, Key Laboratory of Paper-based Functional Materials of China National Light Industry, Shaanxi Provincial Key Laboratory of Papermaking Technology and Specialty Paper Development, Shaanxi University of Science and Technology, Xi’an 710021, China; 3 Department of Natural Sciences/Physics, Åbo Akademi University, Porthaninkatu 3, FI-20500 Turku, Finland; frpetter@abo.fi; 4 Laboratory of Natural Materials Technology, Center for Functional Materials (FunMat), Åbo Akademi University, Porthaninkatu 3, FI-20500 Turku, Finland; Martti.Toivakka@abo.fi

**Keywords:** paper electronics, electrochromic devices, PEDOT:PSS, substrate absorptivity, solid electrolyte

## Abstract

Poly(3,4-ethylenedioxythiophene) doped with the polymer anion poly(styrenesulfonate), PEDOT:PSS, is a common electrochromic material used in the preparation of electrochromic devices (ECDs). In this paper, the PEDOT:PSS doped with a solvent was used both as the electrode and the electrochromic functional layer for fabrication of ECDs on absorptive paper surfaces. The doped PEDOT:PSS dispersion was assessed for the film-forming evenness, sheet resistance and conductivity, and the performance of prepared ECDs for their color contrast and switching dynamics. The ECD performance is discussed in relation to the absorptive characteristics of the substrates. The results indicate that it is feasible to prepare ECDs onto absorptive substrates, despite the partial polymer material imbibition into them. The extent of polymer absorption influences the ECD performance: an increased absorption reduces the color contrast but speeds up the color switching. The electrochemical properties of the used solid electrolyte were found to be crucial for functioning of the ECDs. Insufficient ion transport and associated high resistance led to failure of the devices.

## 1. Introduction

Electrochromic devices (ECDs) are common color modulation devices that utilize regulation of the input electric potential for the color change [1]. Some commercial products such as smart windows, paper-based pixel displays, sunglasses and rear-views mirrors have been developed based on the electrochromic mechanism [2], and even special applications, e.g., infrared camouflage in the military and thermal control in space satellites, have also recently been reported [3,4,5]. Over the past decades, due to the large number of potential end-use applications, various preparation methods of electrochromic devices have attracted much attention by researchers. 

**Figure 1:**
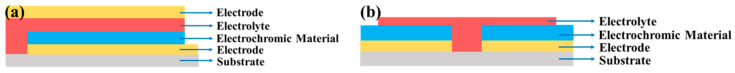
Electrochromic device (ECD) structures: (**a**) Vertical, (**b**) Lateral.

Generally, there are two typical structure configurations that can be chosen for the design of ECDs, a vertical and a lateral one, as shown in Figure 1. The former is composed of an electrode, electrochromic material, electrolyte and counter electrode, which are sequentially deposited on a substrate [6]. The color switch response is relatively fast in this configuration due to the short migration path for ions going through the electrolyte. The color change also occurs evenly and at the same time across the substrate surface. However, the top counter electrode must be transparent and is usually laminated onto the electrolyte surface since depositing the electrode in solution form can dissolve the electrolyte to some extent. A shortcoming of the lamination is that the top electrode can potentially delaminate if the ECD is bent. An ECD with a lateral structure avoids the laminating process by utilizing two electrodes in the same plane covered with electrochromic material and electrolyte. However, the ion transport sideways inside the electrolyte layer can result in a wave effect of coloration and a slow switch response for the color change.

**Figure 2:**
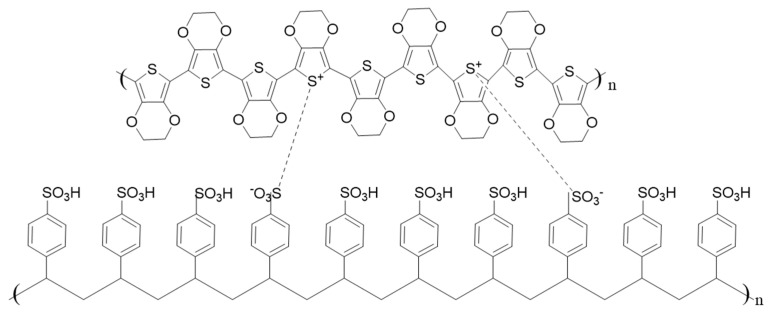
Molecular structure of PEDOT:PSS.

The transparent electrode plays a key role in the performance of an ECD. Indium tin oxide (ITO) has been widely used in the preparation of ECDs due to its high conductivity and transparency. However, the high cost, brittleness, non-environment-friendly preparation process [7] as well as poor adhesion to polymeric materials challenge the applicability of ITO on flexible substrates. Therefore, the replacement of ITO has been a lasting discussion topic in the development of ECDs. For example, metal nanowires [8,9], carbon nanotubes [10], graphene [11] and conductive polymers [12,13] have been suggested as possible substitutes for the ITO electrode. A conductive polymer, poly(3,4-ethylenedioxythiophere), doped with the polymer anion poly(styrenesulfonate) (PEDOT:PSS, Figure 2), has been put forward as a promising transparent electrode material due to such advantages as chemical stability in air and easy processability as an aqueous solution [14]. Films of PEDOT:PSS have high transparency in the visible range, high mechanical flexibility and excellent thermal stability [15,16,17]. However, untreated PEDOT:PSS has very low conductivity, less than 1 S cm^−1^ [18], since the insulated counter ion PSS is needed for sufficient dispersion in water and to improve film forming on surfaces [19]. 

The conductivity of PEDOT:PSS films can be improved with doping by organic solvents, such as ethylene glycol (EG) or dimethyl sulfoxide (DMSO) [20,21]. The main reason for improvement is that the organic solvent in the suspension changes the configuration and morphology of PEDOT:PSS chains. The PSS dissolves into the solvent, increases the intermolecular distances and thereby decreases the Coulomb interaction between PEDOT and PSS. The addition of EG allows the PSS chains to reorganize and the PEDOT nanocrystals to pack together to form a layered structure with a higher order. The addition of EG improved the crystallinity and crystal ordering in the Z direction and enhanced the carrier transport in the XY direction [22].

In addition to being a promising electrode material, PEDOT:PSS is also one of the most common electrochromic materials used in the ECDs. The electrochromic color-switching of PEDOT:PSS results from the electrochemical reaction as follows [23]:

(1) $${\mathrm{PEDOT}}^{+}:{\mathrm{PSS}}^{-}+M^{+}+e^{-}↔{\mathrm{PEDOT}}^{0}+M^{+}{\mathrm{PSS}}^{-}$$

where M^+^ refers to the positively charged ions in the electrolyte, and *e*^−^ presents the electrons in the PEDOT. When applying a voltage to the PEDOT:PSS electrode patterns, the PEDOT in the negatively biased pattern becomes reduced, while the PEDOT in the positively biased pattern becomes oxidized. PEDOT^+^ displays a high concentration of free charge carriers and the optical properties are determined by transitions between bipolaronic states, resulting in a low degree of optical absorption evidenced as very light blue color in the visible wavelength region. In the reduced form, the neutral PEDOT^0^ demonstrates semiconducting properties characterized by a strong optical absorption and dark blue color in the visible wavelength range [24]. 

If the solvent-doped PEDOT:PSS film has sufficient electrical conductivity to drive an ECD and demonstrates electrochromic characteristics, the structure of the ECD can be transformed into a simplified two-layer architecture based on the lateral configuration and the use of the top electrode is avoided. In this case, the constructed ECDs can be built onto non-transparent materials, such as eco-friendly paper material. Traditionally, most ECD designs utilizing PEDOT:PSS as electrochromic material are fabricated on polyethylene terephthalate (PET) and polyethersulfone (PES) films due to their high transparency. However, the petroleum-based plastic substrates are expensive, and have raised environmental concerns during the process of degradation [25]. Therefore, low-cost biodegradable materials such as cellulose-based paper could be potential substrate candidates for flexible ECDs [26,27]. 

While an ECD-based active matrix display on paper-based substrate has been reported [23], the paper substrate was coated with polyester or polyethylene plastic material, thereby converting the cellulose-based paper surface to one comparable to plastic film. The main difference between cellulose-based paper and PET/plastic-coated paper is that the former is porous and absorptive whereas the latter has a sealed surface and is non-absorptive. When applying a solvent-doped dispersion of PEDOT:PSS onto absorptive paper surfaces, the potential permeation into the substrate can influence the color-switching performance of ECDs.

The current work aims at understanding the performance of ECDs fabricated onto absorptive cellulose-based paper using solvent-doped PEDOT:PSS as a common electrode and electrochromic layer, and solid electrolyte to form all solid organic ECDs. The color contrast difference is evaluated and compared to ECDs produced on non-absorptive glass and PET substrates. This work lays ground for expanding the novel ECD development to biodegradable paper-based substrates.

## 2. Experimental

### 2.1. Experimental Materials

Two paper substrates, having either kaolin or anionic silica mineral pigment top-coating, were produced at the Laboratory of Paper Coating and Converting at Åbo Akademi University, Turku, Finland [28,29]. Briefly, both papers contained a 10 g/m^2^ thick sealing layer consisting of platy kaolin pigment (Barrisurf HX, Imerys Minerals Ltd., Par, UK) blended with 50 pph (parts per hundred parts pigment by weight) ethylene acrylic latex (Aquaseal 2077, Paramelt B.V., Heerhugowaard, The Netherlands). On top of this, two different types of porous mineral coatings were applied. The paper substrate labeled “kaolin” had a 5 g/m^2^ top-coating consisting of 70 pph fine platy kaolin (Barrisurf FX, Imerys Minerals Ltd.) and 30 pph fine blocky kaolin (Alphatex, Imerys Minerals Ltd.) blended with 6 pph styrene–butadiene latex (Basonal 2020.5, Basf, Germany). To increase the surface smoothness of the coating, the paper was calendered 3 times with a laboratory scale soft nip calender (DT Paper Science, Turku, Finland). The paper substrate labeled “silica”, had a 1 g/m^2^ thick top-coating consisting of 100 pph anionic silica pigment (Syloid C807, Grace GmbH, Worms, Germany), 20 pph styrene–butadiene latex (DL920, Trinseo Europe GmbH, Samstagern, Switzerland) binder and 5.9 pph synthetic thickener (HPV 56, Dow Europe GmbH, Horgen, Switzerland). Glass and PET (Polyethylene terephthalate, Melinex 506, Pütz GmbH + Co. Folien KG, Taunusstein, Germany were chosen as non-absorptive reference substrates. 

PEDOT:PSS (Clevios PH1000, Heraeus, Hanau, Germany) was obtained as an aqueous dispersion, having a concentration of 1.3% by weight and the weight ratio of PSS to PEDOT of 0.4, corresponding to a molar ratio of 8:1.

Acetone (Ac, Sigma-AldrichFinland Oy, Espoo, Finland), isopropanol (IPA, Sigma-Aldrich Finalnd Oy, Espoo, Finland) and deionized water were used to clean the substrate surfaces, with the exception of the paper surfaces which were used as such. Dimethyl sulfoxide (DMSO, Sigma-Aldrich Finland Oy, Espoo, Finland) and ethylene glycol (EG, Sigma-Aldrich Finalnd Oy) were used as doping solvents to improve the evenness of film formation of PEDOT:PSS and conductivity. Urea (Sigma-Aldrich Finalnd Oy), Choline chloride (ChoCl) (Sigma-Aldrich Finalnd Oy), Poly(sodium-p-styrenesulfonate) (PSSNa) (Merck KGaA, Darmstadt, Germany), Sorbitol (Sigma-Aldrich Finland Oy), Glycerol (Merck KGaA) and HYPODTM 8501 (Dow Chemical Company, Midland, MI, USA) were utilized to prepare the solid electrolyte.

### 2.2. Preparation of PEDOT:PSS Coatings

Three PEDOT:PSS dispersions were prepared: one with pristine PEDOT:PSS as reference (sample A), and two doped with organic solvents, DMSO or EG. The latter two had the following mixing ratios: [PEDOT:PSS]:DMSO = 9:1 [w/w%] (sample B), and [PEDOT:PSS]:EG = 9:1 [w/w%] (sample C). These formulations were stirred for 30 min and then processed with an ultra-sonicator for 10 min. The viscosity of the samples was measured by Brookfield viscometer (CAP 2000+, AMETEK. Inc., Berwyn, PA, USA) using spindle ^#^1 at 20 rpm.

Pre-cut glass substrates were cleaned sequentially using ultra-sonication for 10 min in deionized water, Ac and IPA, and then blow-dried with nitrogen gas. The PEDOT:PSS dispersions were coated onto the cleaned glass slide substrates using a laboratory spin-coater (WS-650MZ-23NPPB, Laurell technology corporation, America) with 1500 rpm under ambient laboratory conditions [30], and dried on a hotplate at 120 °C for 15 min. 

The film forming of the samples was evaluated using an ultra-deep field three-dimensional (3D) digital microscope (KH8700, HIROX, Okinawa, Japan). The film-on-sheet resistance was measured with a four-probe tester (RTS-9, Guangzhou Four Probe Technology, Co. Ltd., Guangzhou, China). Atomic force microscope (AFM, Bruker Corporation, Dalton Asia Pacific, Germany) was employed to measure the thickness of the films and the conductivity *σ*, was then calculated from:

(2) $$\sigma \;=\;\frac{1}{R_{s}\times N}$$

where *N* refers to the thickness, and *R_s_* denotes the sheet resistance.

An optimal formulation, which was selected based on the uniformity and the conductivity of the PEDOT:PSS film, was then spin-coated onto the substrates for the fabrication of ECDs.

### 2.3. Absorption Evaluation of Substrates 

Since the porous top-coatings of the paper substrates are absorptive, their interaction with the PEDOT:PSS dispersion can influence the color-switching performance of the ECDs. Therefore, the sorption characteristics of the substrates were evaluated by Dynamic permeation analyzer (PDA.C 02, Emtec Electronic GmbH, Leipzig, Germany), while the non-absorptive substrates, PET and glass, were used as references. Deionized water was used as the wetting liquid in this test system. The retention of liquids in the substrates was recorded with a Mettler Toledo balance (XS105DU, Mettler-Toledo, LLC, Oakland, CA, USA). Scanning electron microscopy (SEM, SU8100, Hitachi, Ltd., Tokyo, Japan) was used to observe and compare the distribution of PEDOT:PSS dispersion on the paper substrates.

### 2.4. Assessment of Solid Electrolyte and Preparation of ECDs

The characteristics of the electrolyte has an important influence on the performance of ECDs. Three electrolyte candidates based on previous work were considered for fabrication of the ECDs: electrolyte ^#^1 consisting of Sorbitol:ChoCl:8501 (sorbitol and ChoCl 1:1 mol, mixed with 8501 (1:4 w/w%)), electrolyte ^#^2 consisting of PSSNa:H_2_O:Sorbitol:GL (4:7:1:1 w/w/w/w%), and electrolyte ^#^3 consisting of Urea:ChoCl:8501 (Urea and ChoCl 1:1 mol, mixed with 8501 (1:4 w/w%)). The electrolyte solutions were stirred for 2 h to dissolution at ambient temperature and subsequently spin-coated onto ITO-glass electrodes at 1500 rpm and dried for 10 min on the hotplate at 120 °C. The electrolyte film characteristics, impedance, capacitance and cyclic voltammetry (C-V) were measured with a Gamry 600 Impedance Spectrometer (Gamry Instruments, Warminster, PA, USA). The impedance spectra were measured in the frequency range from 0.1 Hz to 0.1 MHz, and the cyclic voltammetry curve was evaluated with a work potential from −2 to 2 V in association with scan rate of 1000 and 5000 mV s^−1^, respectively.

The best performing electrolyte and the PEDOT:PSS dispersion were used to prepare ECDs on different substrates by spin-coating. The CIE1976 color coordinates of ECDs were examined with a portable color reflection spectrometer (X-Rite 528, X-Rite Inc., Grand Rapids, MI, USA) and CIELAB color difference (ΔE^*^) was calculated as:

(3) $$\Delta E^{*}\;=\;{\left[{\left(L_{1}^{*}-L_{2}^{*}\right)}^{2}+{\left(a_{1}^{*}-a_{2}^{*}\right)}^{2}+{\left(b_{1}^{*}-b_{2}^{*}\right)}^{2}\right]}^{1/2}$$

where L*, a* and b* refer to the coordinates along lightness, red-green and yellow-blue color axes respectively, and subscripts 1 and 2 to on and off states of the ECDs.

## 3. Results and Discussion

### 3.1. Optimal PEDOT:PSS Formulation

**Figure 3:**
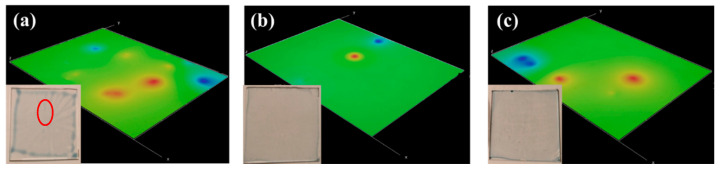
Three-dimensional (3D) digital microscopy and optical images of the spin-coated PEDOT:PSS dispersions on glass. (**a**) Sample A (PH1000), (**b**) Sample B (PH1000 + DMSO), (**c**) Sample C (PH1000 + EG).

The PEDOT:PSS dispersions (Samples A, B and C) were spin-coated on the glass substrate to identify the optimal formulation. 3D digital microscopy and optical images of the coatings are shown in Figure 3. Sample A, the pristine PEDOT:PSS without doping agent, shows a clear star-shaped non-uniformity, marked with red in Figure 3a. Further observing the PEDOT:PSS films through the 3D image, film forming with samples B and C, containing a doping agent, appear more uniform both visually and in the microscope images, especially for sample B. The improved uniformity can be explained not only by the lower viscosity of the samples B (*η* = 36 mPa·s) and C (*η* = 44 mPa·s) compared with the sample A (*η* = 50 mPa·s), but also by the surface tension decrease caused by adding solvents. The surface tensions of DMSO (*γ* = 46.49 × 10^−3^ N/m) and EG (*γ* = 43.6 × 10^−3^ N/m) are lower than the surface tension of PEDOT:PSS (*γ* = 70 × 10^−3^ N/m), and therefore, the surface tension of PEDOT:PSS solution is decreased when doping with the low surface tension solvents. The spin-coating process benefits from low viscosity and low surface tension, both of which promote uniform film formation.

**Figure 4:**
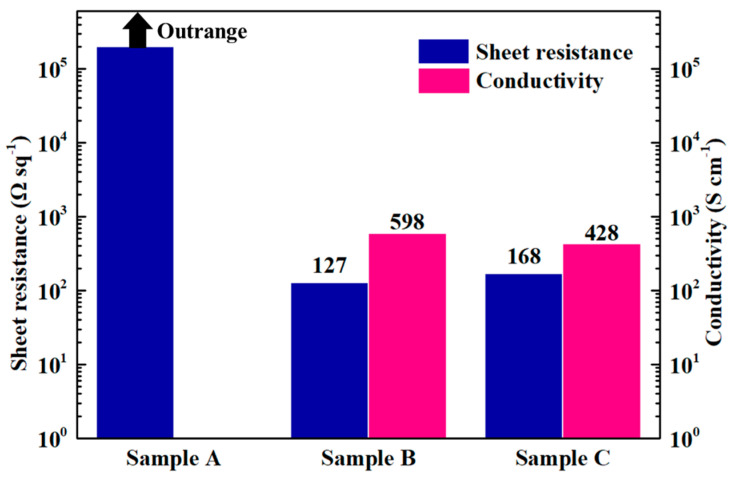
The sheet resistance and conductivity of the PEDOT:PSS dispersions.

Measured sheet resistance and calculated conductivity of the spin-coated PEDOT:PSS films are shown in Figure 4. The pristine PEDOT:PSS dispersion (sample A) has very high sheet resistance, outside the instrument measurement range. Samples B and C, the solvent-doped dispersions, show sheet resistances of 127 and 168 Ω·sq^−1^, respectively. These correspond to conductivities of 598 and 428 S·cm^−1^. The increased conductivity is, as expected, due to the doping with organic solvents [18,31] that reduce the coulomb interaction between PEDOT and PSS chains. 

**Figure 5:**
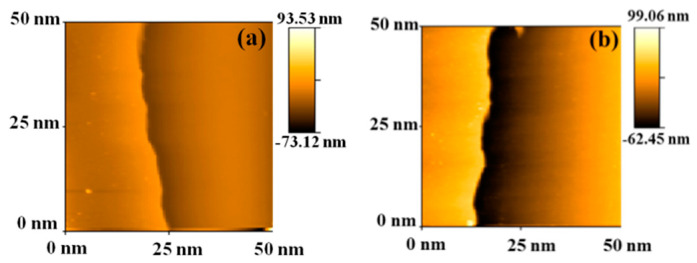
Surface images of the AFM (atomic force microscopy) thickness characterization. (**a**) Sample B, (**b**) Sample C.

The coating thicknesses of sample B and sample C were measured with AFM, as illustrated in Figure 5. Sample B, having the lowest viscosity, produced the thinnest coating of 132 nm, resulting in the 598 S cm^−1^ conductivity. The thickness of the sample C was 139 nm, due to its slightly higher viscosity. Considering the film evenness, the achieved coating thickness and electrical conductivity, the PEDOT:PSS formulation in sample B was chosen for the ECD fabrication on the paper substrates.

### 3.2. Interaction of PEDOT:PSS Formulations with the Substrates

Since PEDOT:PSS formulation can absorb into the porous paper substrate surface, the imbibition and structural characteristics of the substrates were characterized with a dynamic permeation analyzer using a 30 s test time. The ultrasonic measurement provides parameters *C*_i_, *C*_t_ and *T*_95_, which describe the surface characteristics of the substrates. *C*_i_ refers to the ultrasonic energy change from 100% saturation to a corresponding ultrasonic energy level that is defined when the imbibing liquid reaches the coating/material surface. A high value of *C*_i_ indicates a large pore size and roughness of the material surface. *C*_t_ can be used to evaluate the thickness of coating material by considering the imbibition time from the start to a state of liquid passing through a coating layer. A high *C*_t_ value indicates high thickness or slow imbibition. The *T*_95_ value presents the time index when the intensity of ultrasonic energy decreases to 95% of maximum and is suggested to be inversely proportional to the porosity [32]. 

**Figure 6:**
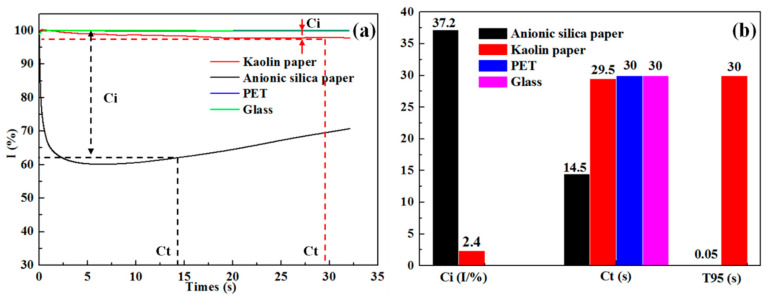
The imbibition characteristics of substrates. (**a**) curves of imbibition based different substrates, (**b**) values of *C*_i_, *C*_t_ and *T*_95_ based different substrates.

The imbibition characteristics of substrates as measured with the dynamic permeation analyzer are shown in Figure 6a. It is clear that the glass and PET substrates do not absorb liquid since their signal levels are maintained at 100% throughout the 30 s measurement. In contrast, both the kaolin and anionic silica-coated papers demonstrate absorption with a decreasing signal strength in time. The ultrasonic measurement parameters in Figure 6b show higher *C*_i_, but lower *C*_t_ and *T*_95_ values for the silica paper in comparison to the kaolin-coated paper. This suggests that the former has higher surface roughness, larger pores, thinner coating and higher porosity. The large pores and high porosity can lead to a high penetration of PEDOT:PSS dispersion into the coating, thereby reducing the amount remaining on the substrate surface. Therefore, we hypothesize that more particles of PEDOT:PSS dispersion are trapped into pores, not onto the surface of anionic silica-coated paper.

The amounts of PEDOT:PSS remaining on the substrate surfaces when applied with spin-coating at constant speed (Table 1) agree with the measured imbibition characteristics. The non-absorptive smooth substrates, PET and glass, retained 0.11–0.12 mg·cm^−2^, whereas the kaolin-coated paper retained 0.17 mg·cm^−2^ and the highly porous anionic silica coating retained the highest amount, 0.23 mg·cm^−2^. 

**Figure 7:**
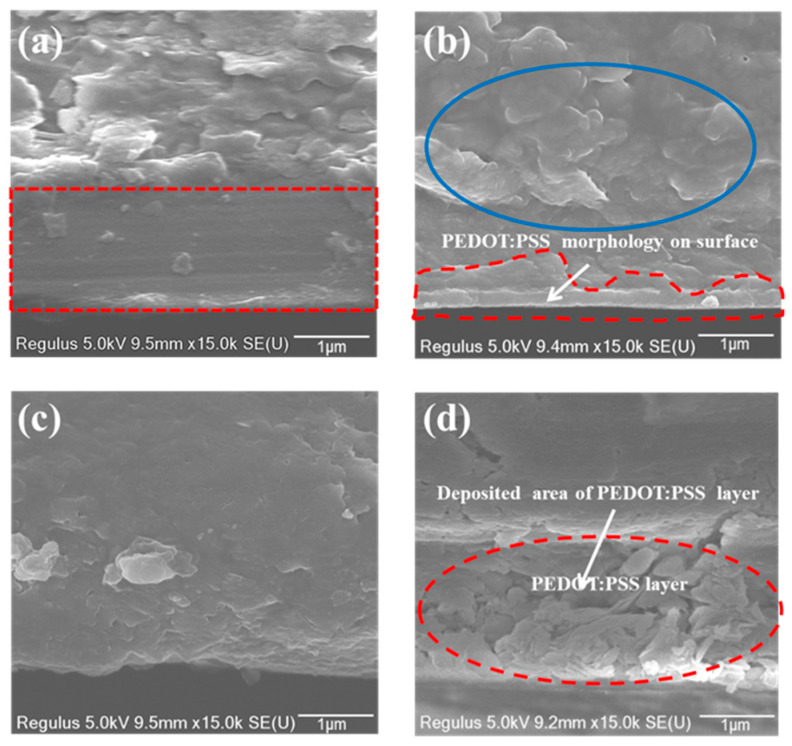
Cross-section SEM (scanning electron microscopy) images of paper substrates and the retained PEDOT:PSS dispersion on them. (**a**) Anionic silica-coated paper, (**b**) PEDOT:PSS dispersion-coated surface of anionic silica-coated paper, (**c**) Kaolin-coated paper, (**d**) PEDOT:PSS dispersion-coated surface of Kaolin-coated paper.

Figure 7 shows the SEM images of uncoated and PEDOT:PSS spin-coated paper surfaces and cross-sections. Comparing the images, it is clear that the kaolin coating (Figure 7c) is smoother than the anionic silica coating (Figure 7a) as predicted in the imbibition test. Due to the large pores in the silica coating, most of the PEDOT:PSS seems to have penetrated deep into the coating, leaving only a small amount on the surface, as indicated by the blue circle in Figure 7b. The kaolin coating, which has closed surface morphology, appears to retain much more PEDOT:PSS on its surface, evidenced as graininess in the red circle in Figure 7d. The polymer interaction with the substrate surfaces as observed in the SEM images agrees with the imbibition and retention measurements. Of course, the amount of PEDOT:PSS dispersion remaining on the surface can also influence the performance of prepared ECDs, which will be discussed in the following.

### 3.3. Performance Evaluation of the Electrolytes

**Figure 8:**
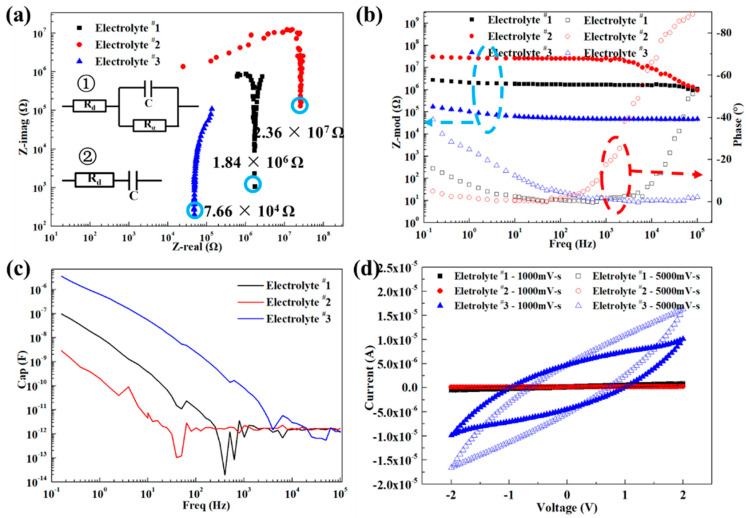
Electrochemical characterization. (**a**) Nyquist plot, (**b**) Bode plot, (**c**) Capacitance, (**d**) Current–Voltage plot.

The electrolyte for preparation of ECDs on absorptive substrate needs to be in solid state for avoiding the damages for substrate in liquid/gel state while maintaining sufficient ion mobility to provide adequate performance in end-use. In the current work, properties of three solid-state electrolyte candidates were examined and characterized by impedance spectroscopy. The characteristics are compared in Figure 8 using the Nyquist plot, Bode plot and capacitance. Using a dedicated ZSimpwin software to evaluate the impedance spectroscopy data, we fit the curves using one of two different equivalent circuits, and then calculated the values presented in Figure 8. Electrolytes ^#^1 and ^#^2 are fitted to circuit ①, and electrolyte ^#^3 to circuit ②, in Figure 8a. The values obtained from the fits, *R*_d_ (internal resistance of the electrolyte solution), *C* (capacitance) and *R*_α_ (polarization resistance of the charge transfer process), are listed in Table 2. The internal resistance of electrolyte ^#^3 (7.66 × 10^4^ Ω) is lowest, almost 2 to 3 orders of magnitude lower than those for the electrolytes ^#^1 (1.84 × 10^6^ Ω) and ^#^2 (2.36 × 10^7^ Ω). Figure 8a, b indicate that the low internal resistance assists the ions and leads to a high mobility state in the electrolyte. Similarly, Figure 8c shows that at low frequencies, the electrolyte ^#^3 has the highest capacitance (*C* = 3.15 × 10^−6^ F), when compared to the electrolytes ^#^1 (*C* = 1.13 × 10^−7^ F) and ^#^2 (*C* = 3.18 × 10^−9^ F). 

From the C-V graph in Figure 8d, it can be seen that the electrolyte ^#^3 supplies a larger charge capacity under different scan rates, showing a more powerful ability of charge storage and transfer performance compared to electrolyte ^#^1 and electrolyte ^#^2. While the performance of the electrolytes depends mostly on their electro-chemical properties, it is also influenced by the electrolyte viscosity, which controls film forming when coated or printed to produce a device. As shown in Table 3, the viscosity of electrolyte ^#^3, *η =* 107 mPa·S, is less than that of electrolyte ^#^1 (*η =* 142 mPa·S) and electrolyte ^#^2 (*η =* 265 mPa·S). This is a result of the high viscosity of Sorbitol and Glycerol, which were used to prepare electrolytes ^#^1 and ^#^2, respectively. 

Considering the properties of the internal resistance (ion mobility), capacitance and C-V curves of the electrolytes, it is inferred that the electrolyte ^#^3 can demonstrate better performance in the preparation of ECDs in comparison to the electrolytes ^#^1 and ^#^2.

### 3.4. Performance of Prepared ECDs

For the preparation of ECDs, the optimal PEDOT:PSS dispersion (Sample B) was spin-coated as a common electrode and electrochromic layer on the two absorptive substrates (kaolin-coated paper and anionic silica-coated paper) and the non-absorptive PET and glass. Then, electrolytes ^#^1, ^#^2 and ^#^3 were spin-coated on top to produce a simple lateral ECD architecture, shown in Figure 1b. Color changes of ECDs during 120 s were evaluated using a working potential of 4 V for observing a whole change. The electrolytes ^#^1 and ^#^2 did not display any color switching, which is attributed to their high internal resistance and low ion mobility. 

**Figure 9:**
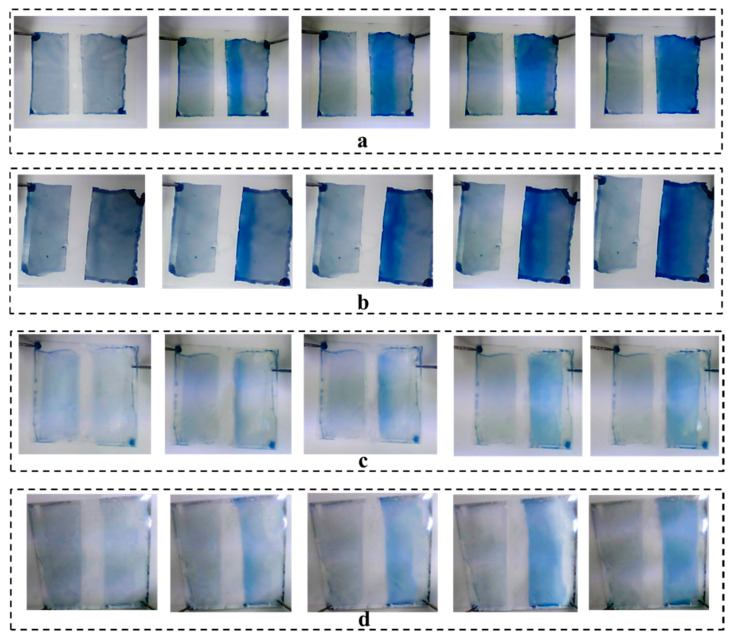
The recorded color change and the waving effect of the ECDs fabricated on different substrates: (**a**) anionic silica-coated paper, (**b**) kaolin-coated paper, (**c**) PET, (**d**) Glass slide.

**Figure 10:**
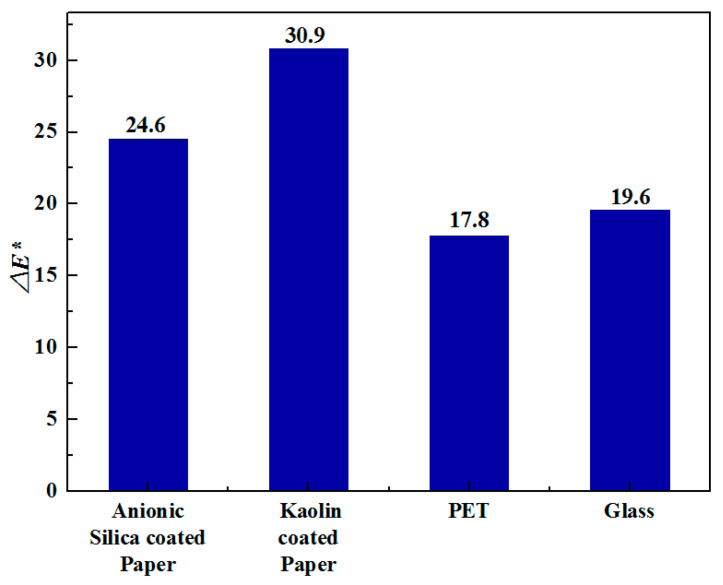
The color contrast range, ΔE^*^, of the devices fabricated on the different substrates.

Although the internal resistance of electrolyte ^#^3 is also not very small, it could still initiate a color change already below the applied 4 V potential. Figure 9 shows the color switching and the waving effect in time for the ECDs fabricated with the electrolyte ^#^3 on the four substrates. Figure 10 quantifies the color switching at 120 s with the color contrast difference, ΔE^*^. The best performance is obtained on the absorptive substrate of kaolin-coated paper, reaching a color contrast ΔE^*^ as high as 30.9. On the anionic silica-coated paper, the ECD provides a contrast of 24.9. The differences in the color switching can be ascribed to the amount of PEDOT:PSS dispersion staying on the substrate surfaces. The low amounts of PEDOT:PSS on PET and glass substrates result in color contrast change of 17.8 and 19.6, respectively. A high amount and a thick layer of PEDOT:PSS can increase the time needed for the color switching [33], as seen in the Figure 9. For example, the ECDs on kaolin-coated paper accomplished a complete color change in 120 s, whereas on the anionic silica-coated paper, only 90 s is needed. On PET and glass substrates, due to the thin electrochromic layer, a relatively short time is required to finish the color switching of the whole area.

## 4. Conclusions

In this paper, ECDs with a lateral architecture were fabricated on different absorptive substrates using PEDOT:PSS both as the electrode and the active electrochromic functional layer, and solid electrolyte. The conductivity of the PEDOT:PSS dispersion film was enhanced by solvent-doping. Doping with DMSO was found to provide slightly better performance than with EG. 

Three solid electrolytes were considered by evaluating their electrochemical properties, including the internal resistance (ion mobility), capacitance, C-V performance curve and viscosity. Electrolyte ^#^3, consisting of Urea:ChoCl:8501 solution, showed superior performance compared to the two other electrolytes, which suffered from high internal resistance and therefore could not initiate a color change for the ECDs. The internal resistance of the electrolyte should be a key consideration to fabricate the ECDs.

Using the best performing electrolyte and the doped PEDOT:PSS dispersion, functioning ECDs were fabricated on the two absorptive papers. A higher color contrast change but slower switching was observed on the kaolin-coated paper compared to the anionic silica-coated paper. The former has a closed coating structure, which retained a high amount of the spin-coated PEDOT:PSS on the paper surface, which then contributed to the observed color switching behavior. As a conclusion, it is feasible to manufacture ECDs directly on absorptive substrates, although imbibition of the active polymer layer into the porous surface should be minimized for improved device performance. In order to maintain the functional inks, such as PEDOT:PSS, on the surface of the absorptive paper, the coating formulation could be optimized through use of a combination of platy kaolin mineral pigment particles and natural binders such as starch or nanocellulose.

## Figures and Tables

**Table 1:** The retention of PEDOT:PSS on different substrates.

Substrate	Grammage before Coating/mg·cm^−2^	Coated Area/cm^2^	Weight of Substrate with Coating/mg	Grammage after Coating/mg·cm^−2^	Change in Grammage/mg·cm^−2^
Anionic Silica-coated paper	14.42	2.52	36.92	14.65	0.23
Kaolin-coated paper	13.43	2.99	40.67	13.60	0.17
PET	14.32	2.35	33.93	14.44	0.12
Glass	250.96	5.63	1413.52	251.07	0.11

**Table 2:** The values of R_d_, C and R_α_.

Electrolyte	R_d_/Ω	C/F	R_α_/Ω
electrolyte ^#^1	1.84 × 10^6^	1.13 × 10^−7^	4.795
electrolyte ^#^2	2.36 × 10^7^	3.18 × 10^−9^	13.625
electrolyte ^#^3	7.66 × 10^4^	3.15 × 10^−6^	-

**Table 3:** The viscosity of electrolyte and key elements in electrolyte.

Material	Viscosity/mPa·S	Mass Fraction/%	Temperature/°C
Sorbitol	900	80	25
Glycerol	1500	99	25
Urea	1.55	50	25
electrolyte ^#^1	142	-	25
electrolyte ^#^2	265	-	25
electrolyte ^#^3	107	-	25
